# Synthesis, characterization, and mercury adsorption properties of hybrid mesoporous aluminosilicate sieve prepared with fly ash

**DOI:** 10.1016/j.apsusc.2013.02.116

**Published:** 2013-05-15

**Authors:** Minmin Liu, Li-an Hou, Beidou Xi, Ying Zhao, Xunfeng Xia

**Affiliations:** a School of Environmental Science and Engineering, Tongji University, 1239 Siping Road, Shanghai 200092, PR China; b China Research Academy of Environmental Science, Beijing 200012, PR China

**Keywords:** MCM-41, Fly ash, Mercury adsorption, Isotherms, Mesoporous sieve

## Abstract

A novel hybrid mesoporous aluminosilicate sieve (HMAS) was prepared with fly ash and impregnated with zeolite A precursors. This improved the mercury adsorption of HMAS compared to original MCM-41. The HMAS was characterized by X-ray diffraction (XRD), nitrogen adsorption–desorption, Fourier transform infrared (FTIR) analysis, transmission electron microscopy (TEM) images and ^29^Si and ^27^Al magic angle spinning nuclear magnetic resonance (MAS NMR) spectra. These showed that the HMAS structure was still retained after impregnated with zeolite A. But the surface area and pore diameter of HMAS decreased due to pore blockage. Adsorption of mercury from aqueous solution was studied on untreated MCM-41and HMAS. The mercury adsorption rate of HMAS was higher than that of origin MCM-41. The adsorption of mercury was investigated on HMAS regarding the pH of mercury solution, initial mercury concentration, and the reaction temperature. The experimental data fit well to Langmuir and Freundlich isotherm models. The Dublin–Radushkevich isotherm and the characterization show that the mercury adsorption on HMAS involved the ion-exchange mechanisms. In addition, the thermodynamic parameters suggest that the adsorption process was endothermic in nature. The adsorption of mercury on HMAS followed the first order kinetics.

## Highlights

► Adsorption of mercury. ► Impregnation of A zeolite on MCM-41. ► Prepared with fly ash.

## 1 Introduction

Presence of mercury in water bodies is an indicative of water contamination caused by municipal or industrial wastewater. Many industrial plants such as textile factories, fertilizer industry, mining facilities and tanneries discharge wastewater containing many kinds of heavy metals including mercury. Many methods have been carried out to remove mercury from wastewater. Carbon adsorption, ion exchange, chemical precipitation, membrane filtration and photocatalysis reduction have been conventionally applied to treat heavy metal wastewater [1]. The adsorption process is more suitable than other methods for the mercury removal from wastewater regarding technique, economy and health.

Mesoporous materials with ordered pore structure, large surface area have shown promise for applications ranging from air to water purification [2]. These materials are considered to have good potential for adsorption/separation applications because of regular hexagonal structure, uniform pore distribution, large surface area and large pore volume [3,4]. Untreated or modified mesoporous silica has been applied to different pollutants adsorption. For example, MCM-41 was used to remove nitrobenzene, phenol, *o*-chlorophenol and divalent metal cations from wastewater [5,6]. MCM-41 was also used to remove volatile organic compounds (VOCs) from indoor air [7–9]. In addition, modified mesoporous silicas were verified to be good adsorbents for the removal of heavy metals from solutions [10–14]. Modified MCM-41 materials were applied to adsorb anionic dyes, mercury [15,16].

Some studies showed that the assembly of MCM-41 with nanosized zeolite precursors not only significantly improved its hydrothermal stability [17–20] but also improved the adsorption of heavy metals on MCM-41 from aqueous solution [21–23]. However, limited studies have focused on synthesizing hybrid mesoporous sieve with fly ash.

In this paper, investigations have been carried out to study the synthesis, characterization and mercury adsorption on HMAS. HMAS was synthesized with fly ash and impregnated with performed zeolite A precursors. The efficiency of mercury adsorption on HMAS was studied regarding the pH of mercury solution, initial mercury concentration, and the reaction temperature. In addition, the mechanisms of mercury adsorption were discussed according to the isotherms, kinetics and thermodynamics models.

## 2 Experimental

### 2.1 Materials and chemical reagents

Cetyltrimethylammonium bromide (CTAB, 98%) was supplied by Aldrich (U.K.). Sodium silicate (99%), sodium aluminate (99%), sodium hydroxide (99%), hydrochloric acid (99 wt.%)was provided by Fisher Scientific. All the glassware were soaked in 5% HCl overnight and cleaned with deionised water before use. Two types of coal fly ash were collected from two different coal-fired power plants. The fly ashes samples were labeled as follows: ZF-1 from the Linhe Power Plant in Inner Mongolia; ZF-2 from the Baotou Power Plant in Inner Mongolia.

### 2.2 Synthesis of HMAS

#### 2.2.1 Preparation of precursors of A zeolite

The precursors of A zeolite were prepared as previously reported in the synthesis of A zeolite [24]. The main process is as follows: the chemicals Na_2_SiO_3_·9H_2_O, NaOH, and NaAlO_2_ with molar ratio of Na_2_O:Al_2_O_3_:SiO_2_:H_2_O equal to 3.165:1:1.926:128 were mixed in distilled water in the boiling state and stirred for 1 h. After that, aged at 298 K in a static state for 24 h to form the precursors of A zeolite.

### 2.3 Synthesis of HMAS

**Fig. 1:**
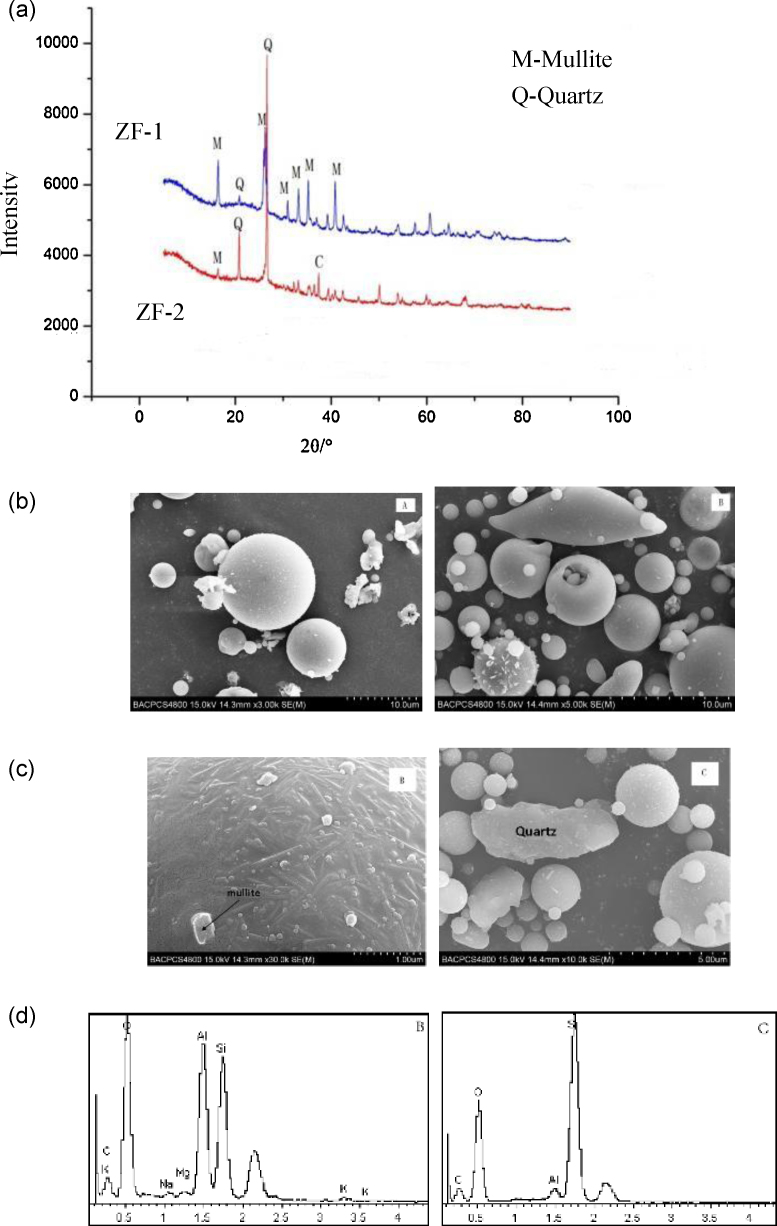
(a) Fly ash XRD spectra; (b) SEM images of fly ashes: ZF-1 (A), ZF-2 (B); (c) Morphology of main phases in fly ashes: mullite (B), quartz (C); (d) EDX chemical analysis determined in main fly ash phases: mullite (B), quartz (C).

SEM images in Fig. 1 show the fly ash morphology. Two kinds of fly ashes were composed mainly of spherical hollow particles. Other phases with different shape, size and texture were identified by SEM and analyzed by EDX. Particles in fly ash ZF-1 with irregular agglomerations shown in Fig. 1c and d were identified with high alumina and silica contents. Quartz was present as irregular particles in fly ash ZF-2 with high silicon and oxygen content (Fig. 1d).

According to the Fig. 1a and Table 1, they are shown that Fly ash ZF-2 was found to be fly ash with high amorphous material and low quartz and mullite contents. High amorphous SiO_2_ and Al_2_O_3_ content and low quartz and mullite content are two important characteristics that indicate high fly ash reactivity in direct conversion processes, such as alumina and silica extraction for synthesizing molecular sieves. In addition, Fly ash ZF-1 contains more Mullite than ZF-2. Thus, ZF-1 and ZF-2 were both pretreated by 60% of 12 M HCl in 60% water bath for 10 h and the weight ratio of fly ash to HCl was 1:10.

Table 2 presented the silica and alumina contents of ZF-1 and ZF-2 after acid pretreatment. From Table 2, it can be known that the amorphous alumina contents of fly ashes were decreased and the silica contents were not changed. Thus, the molar ratios of silica to alumina were enhanced after acid treatment of fly ash.

Then, after acid treatment of fly ash, fly ash was treated by alkali treatment. The specific process was as follows: the weight ratio of 2 M NaOH to fly ash was 3:1 and it was heated to boil for 2 h. Then the silica and alumina contents of the solution were examined by ICP. As shown in Table 2, after acid treatment, the ZF-1 contained both of silica and alumina. It is because mullite is composed of silica and alumina and mullite content of ZF-1 was 30.69%. However, ZF-2 mainly contained silica after acid treatment. It is because the mullite content of ZF-2 was less than 4% and ZF-2 was mainly composed of the quartz and amorphous silica. So alkali treatment can extract silica and alumina for synthesizing mesoporous sieves.

From Table 3, the volume ratio of ZF-1 alkali solution to ZF-2 alkali solution was adjusted to the optimum molar ratio of silica to alumina. In the process of synthesizing mesoporous adsorbents, the molar ratio of (SiO_2_ + Al_2_O_3_):NaOH:C_16_TMABr:H_2_O was equal to 1:0.24:0.12:100 and the molar ratio of SiO_2_:Al_2_O_3_ was equal to 20:1. The 70 mL of C_16_TMABr aqueous solution containing 4.37 g C_16_TMABr was mixed with 30 mL of the ZF-1 alkali solution, 10 mL of the ZF-2 alkali solution, and 0.4 g sodium aluminate to form the original solution. 1% of weight percentage of A zeolite precursors were added to the original solution. Then the pH value of the mixture solution was adjusted to 10.5 with hydrochloric acid, resulting in a gel, after 1 h continuous stirring. The gel was transferred into a 200 mL Teflon-lined stainless autoclave and crystallized at 378 K for 48 h. After crystallization, the autoclave was cooled to room temperature naturally. Then the product was filtered out and dried at 378 K for 10 h. Eventually, the powder was calcined in air at 823 for 4 h to remove the surfactant, using a ramping rate of 2 K/min. Finally, the white powder was obtained.

### 2.4 Characterization and analysis

#### 2.4.1 Fly-ash characterization

Fly ash chemical composition was determined on a Philips type PW2404 fluorescence X-ray spectrometer. The fly ash samples were homogenized and dried for 24 h at 105 °C. Mineralogical compositions were determined using an X-ray diffractometer D8 advance powder diffractometer with Cu Kα radiation. Diffraction patterns were collected at 5–90° using Cu Kα radiation with a step size of 0.028°, and CaF_2_ as internal standard. The morphology of different crystalline phases identified by XRD was inferred by combining the results obtained from scanning electron microscopy (SEM) and energy dispersive X-ray (EDX). Samples were coated with gold and analyzed with a Hitachi S-4800 Scanning Microscope.

#### 2.4.2 HMAS characterization

The quantitative evaluation of the structural units was obtained by small-angle X-ray scattering (SAXS) measurements. A Philips X’pert powder diffractometer system with Cu Kα (*λ* = 1.541 Å) radiation was used for X-ray studies. XRD analysis was performed from 1.5° to 10.0°. The wide-angle X-ray scattering of HMAS was measured using an X-ray diffractometer D8 advance powder diffractometer with Cu Kα radiation. Diffraction patterns were collected at 5–90° using Cu Kα radiation with a step size of 0.028°, and CaF_2_ as internal standard. The Brunauer–Emmett–Teller (BET) specific surface area was calculated using the standard BET method for adsorption data in the relative adsorption range from 0.05 to 0.2. The total pore volume was estimated on the basis of the amount of nitrogen adsorbed at a relative pressure (*P*/*P*_0_) of ca. 0.99. The pore size distribution (PSD) was determined using the Barrett–Joyner–Halenda (BJH) method applied to the adsorption branch of the isotherm. Infrared spectra of all samples were obtained in KBr pellets in the 4000–400 cm^−1^ region with a resolution of 4 cm^−1^, by accumulating 64 scans using an ATI Mattson FTIR spectrophotometer. Transmission electron microscopy (TEM) images were taken on an H-8100 transmission electron microscopy operated at 200 kV. The concentration of mercury(II) in water was determined by inductivity coupled plasma atomic emission spectrometry (ICP-AES) using a PerkinElmer Optima 5300DV instrument (PerkinElmer, UK) at the an RF power of 1300 W and with plasma, auxiliary and nebulizer argon gas flows of 15, 0.2 and 0.75 L min^−1^ respectively, and a pump flow rate of 1.5 mL min^−1^. The solid states ^29^Si and ^27^Al magic angle spinning nuclear magnetic resonance (MAS NMR) spectra were recorded on a Varian Unity Inova 400M spectrometer at 59.584 MHz and 78.155 MHz, using 1.5 μs and 0.3 μs pulse length, 3 s and 1 s recycle delays, and a spinning rate of 5 kHz and 7 kHz, respectively. ^27^Al chemical shifts were measured relative to Al (H_2_O)_6_^+3^.

### 2.5 Point of zero charge (pH_ZPC_)

If the pH_ZPC_ is higher than the pH of the solution, the acidic solution donates more protons than hydroxide groups. Thus, the adsorbent surface is positively charged. If the pH is above pH_ZPC_, the surface is negatively charged.

The pH_ZPC_ of samples was determined by the batch equilibration technique [22]. The process is as follows: 10 mg of the sample in 20 mL 0.1 M NaCl solution. The initial pH value (pH_*i*_) of NaCl solution was adjusted from 1 to 7 by addition of 0.1 M HCl and 0.1 M NH_4_OH. The suspension reached equilibration at 25 °C for 24 h for stirring. Then it was filtered and the final pH value (pH_*f*_) was measured. The pH_ZPC_ was obtained from the plot of pH_*i*_ and pH_*f*_ values. The same procedure was carried out in 0.01 M NaCl solution.

### 2.6 Adsorption experiments

Batch adsorption experiments were conducted to test the effect of different parameters such as the pH of mercury solution, initial mercury concentration and reaction temperature on mercury adsorption by HMAS. The mercury adsorption experiments were all conducted in distilled water. Approximately 10 mg HMAS was suspended in 10 mL of solution containing 2–16 mg/L mercury and the solution was stirred (300 rpm) for approximately 2 h at 303 ± 2 K. At the end of the adsorption process the adsorbent was filtered through the 0.45 μm membrane filter and the residual solution concentration of mercury was analyzed by ICP-AES. In order to reduce measurement errors in all the experiments, the mercury concentration of each equilibrium solution sample was measured in triplicates. In addition, the average value was used to calculate the equilibrium concentration based on a standard calibration curve, whose correlation coefficient square was 0.9999. The experimental error was observed to be within ±2%.

The specific amount of mercury adsorbed was calculated using Eq. (1) [25] as follows:

(1) $$q_{\text{e}}=\frac{(C_{0}-C_{\text{e}})\times V}{W}$$

where $q_{\text{e}}$ is the adsorption capacity (mg/g) in the solid at equilibrium; $C_{0}$ and $C_{\text{e}}$ are the initial and equilibrium concentrations of solution (mg/L), respectively; *V* is the volume of the aqueous solution (*L*) and *W* is the mass (g) of adsorbent used in the experiments.

## 3 Results and discussion

### 3.1 Characterization of mesoporous adsorbent

#### 3.1.1 X-ray diffraction

**Fig. 2:**
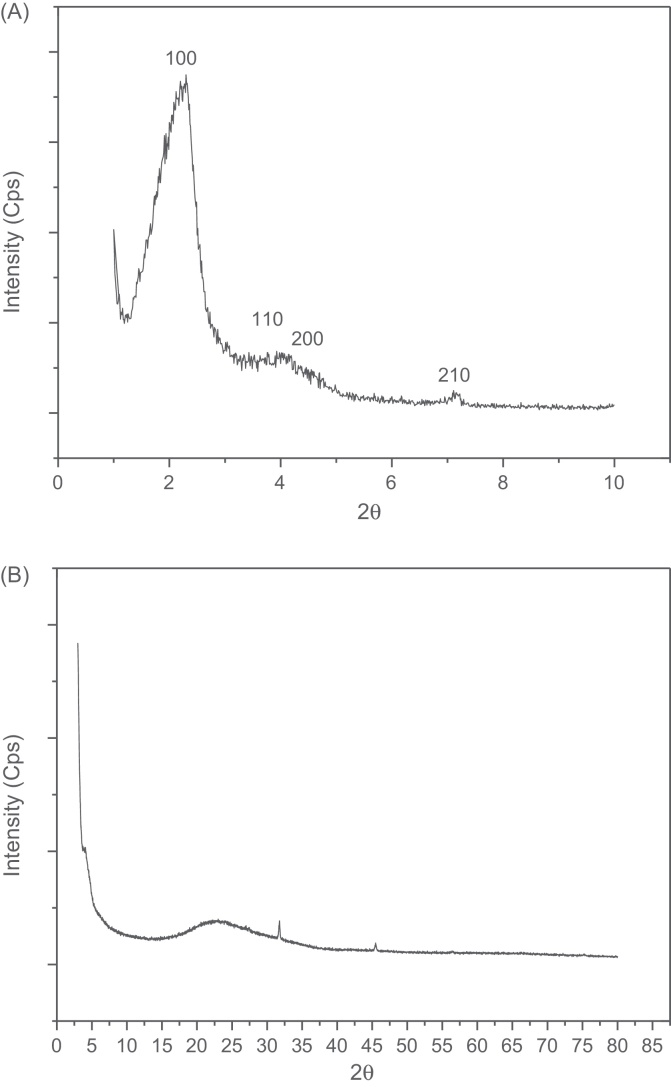
Small angle XRD pattern (A) and wide angle XRD pattern (B) of HMAS.

The small angle and wide angle XRD patterns of the molecular sieve are shown in Fig. 2. The small angle XRD pattern of the sample is presented in Fig. 2a. It demonstrated that the molecular sieve has the typical long-range ordered hexagonal mesoporous structure. The structure can be verified by the observation of four distinct diffraction peaks indexed as (1 0 0), (1 1 0), (2 0 0), and (2 1 0) in the low 2*θ* region. In addition, the interplanar distance of the sample is 5.29 nm. The hexagonal unit cell parameter *a*_0_ = 2*d*_100_/1.732 of the sample is 6.11 nm.

The wide angle XRD pattern of HMAS does not show any obvious diffraction peak. It suggests that the zeolite A units were dispersed in the pore wall of the sample.

#### 3.1.2 N_2_ adsorption–desorption isotherm

**Fig. 3:**
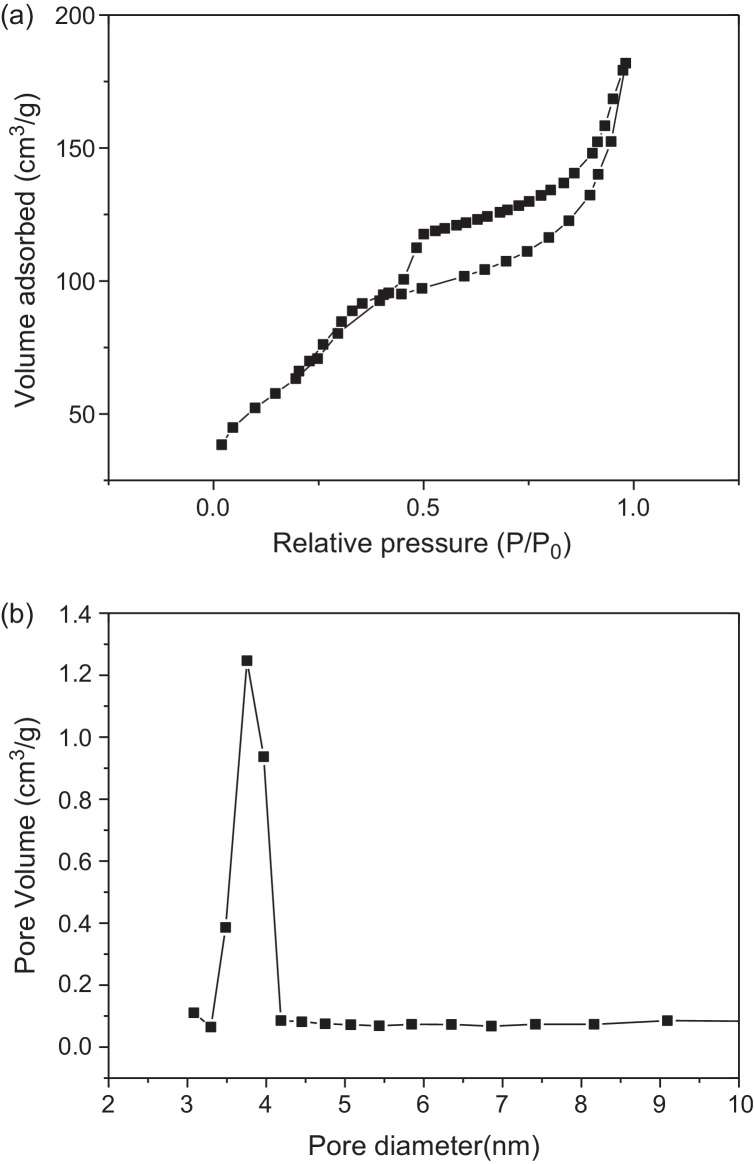
N_2_ adsorption–desorption isotherm of HMAS (a); BJH pore size distribution curve of HMAS (b).

Nitrogen adsorption–desorption isotherms of the sample is illustrated in the Fig. 3a. The sample has type IV classiﬁcation isotherms, which is the characteristic of adsorption of mesoporous materials MCM-41. The presence of a sharp sorption step in adsorption curves, near to a 0.5 value of *P*/*P*_0_ indicates that the solid possesses a well-deﬁned array of regular mesopores. There is a deep inflection of the molecular sieve between relative pressure *P*/*P*_0_ = 0.5 and 1.0. It demonstrated that it has the uniformity of the pores distribution due to characteristics of capillary condensation [26]. The fact that the isotherm for the HMAS is similar in shape to that of MCM-41, suggests that the zeolite A should be dispersed uniformly throughout the pores. There are microporous structures in sample because the slope is at very low relative pressure. The BET surface area of molecular sieve is around 485 m^2^/g, and the BJH mean pore diameter is 4.60 nm. The formula *T* = *a*_0_ − *d*_BJH_ calculates the pore wall thickness, and the *a*_0_ is the hexagonal unit cell parameter. The *d*_BJH_ denotes the mean pore diameter. Thus, the pore wall thickness is 1.51.

The pore distribution of molecular sieve is presented in Fig. 3b. The result verified that the pores of 4 nm diameter occupied most part of pore volume of the sample. Additionally, it showed that pores of the sample are very uniform.

#### 3.1.3 Fourier transform infrared (FTIR) spectroscopy

**Fig. 4:**
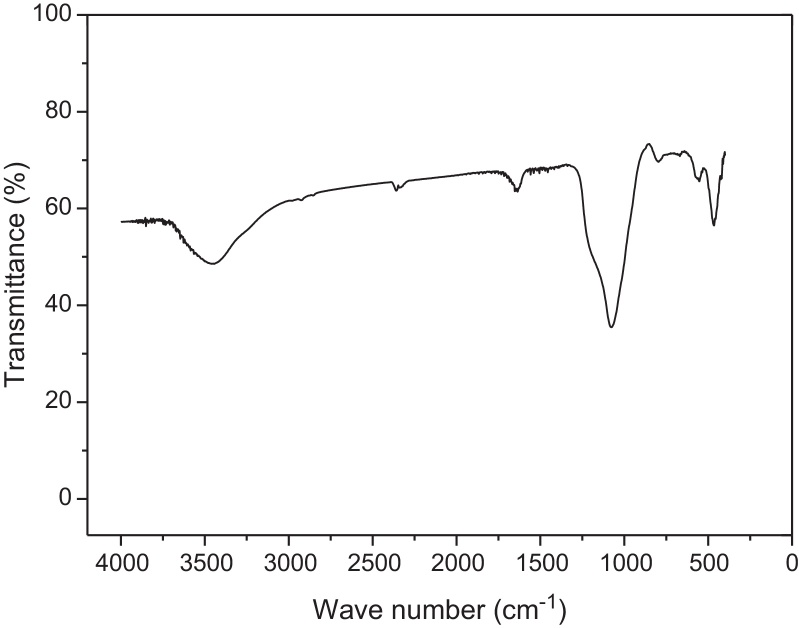
FT-IR spectrum of HMAS.

The FTIR spectrum of the molecular sieve is illustrated in Fig. 4. The vibrational band around 465 cm^−1^ means the zeolite A is distributed in the silica framework of the molecular sieve and it is assigned as characteristic of 5-ring and 6-ring T–O–T (T can be Si or Al) in the pore walls [27]. The vibrational bands at 1087 and 465 cm^−1^ are attributed to the characteristic silica framework in MCM-41 [28]. The band around 1634 cm^−1^ is attributed to the characteristics of water molecules inside the framework, and the bands around 3445 cm^−1^ correspond to OH— groups from water molecules [29]. This illustrated that the sample is hydrophilic and it adsorbs some water when it is exposed to the air.

#### 3.1.4 ^29^Si and ^27^Al MAS-NMR

3.1.4

**Fig. 5:**
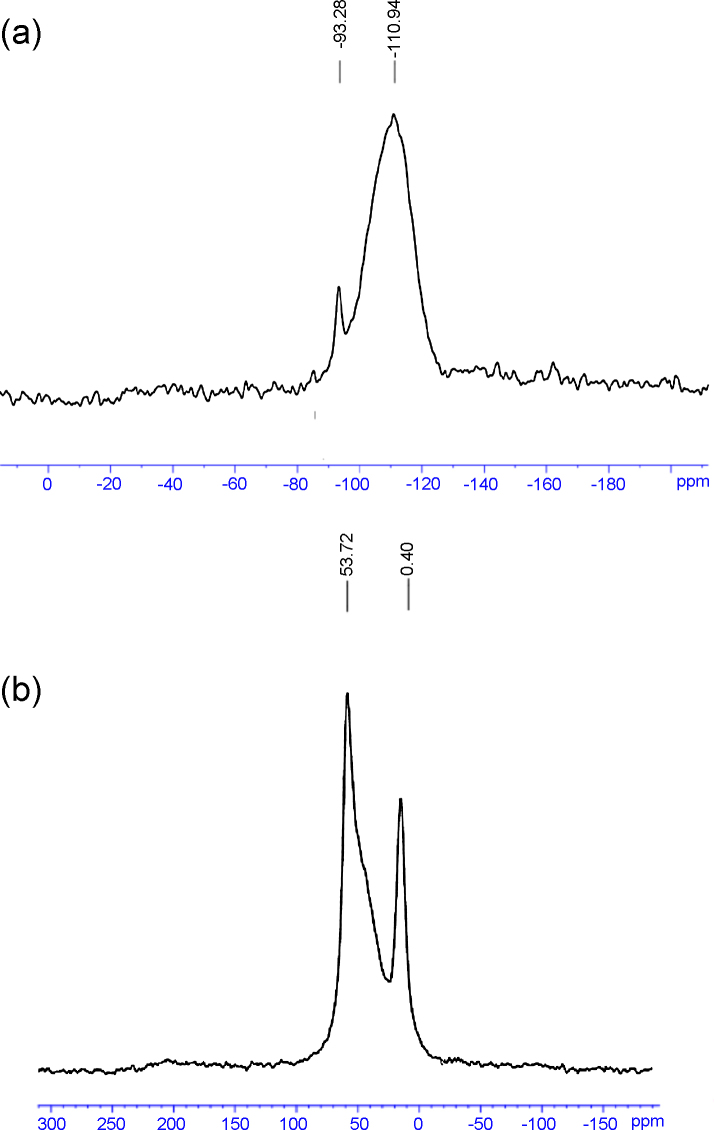
^29^Si MAS-NMR (a) and ^27^Al MAS-NMR (b) spectra of HMAS.

The ^29^Si and ^27^Al MAS-NMR spectrum of HMAS are presented in Fig. 5. From the ^29^Si MAS-NMR spectra of the sample, ^29^Si MAS-NMR spectra contain two signals at −93 and −110 ppm. In addition, the signal at −93 ppm and the little broad (right side) signal at −110 ppm can be decomposed three resonance peaks. Three peaks mean three structures of silicon atoms from left to right side. These structures are indexed as *Q*^2^, *Q*^3^, and *Q*^4^ according to the *Q*^*n*^ = Si [*n*Si, (4 − *n*) OH], or Si [*n*Si, (4 − *n*) Al], *n* = 1–4 [24]. *Q*^4^:*Q*^3^ of HMAS was about 10.36.

Fig. 5b shows the Al coordination state of HMAS sample measured by the solid-state ^27^Al MAS NMR. Two NMR peaks at 53.72 ppm and 0.40 ppm are observed in Fig. 5b. The peak at 53.72 ppm is attributed to aluminum species bound to four —O—Si groups covalently in tetrahedral framework and a portion of silicon in the framework was replaced by aluminum. In addition, the peak at 0.40 ppm is attributed to aluminum species in hexahedral framework and these aluminum species were extra-framework. Aluminum species in hexahedral framework generate strong acid sites and make HMAS have ion exchange capacity. As shown in Fig. 5b, some aluminum species of HMAS are in tetrahedral framework and other aluminum species are in hexahedral framework of zeolite A units which were dispersed in the pore wall of HMAS.

#### 3.1.5 Transmission electron microscope (TEM) image

**Fig. 6:**
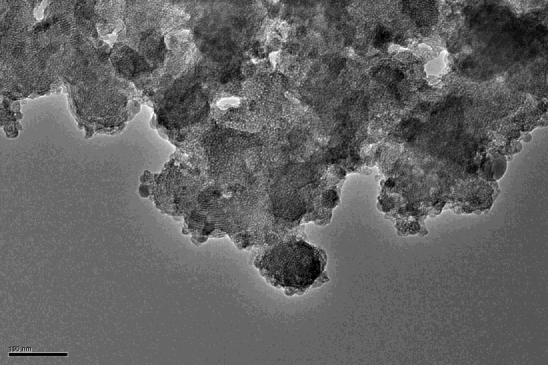
TEM image of the HMAS.

The TEM image of the molecular sieve is shown as Fig. 6. The TEM image of the sample confirmed that the material possesses uniform pores. The mesoporous pores distributed uniformly in the sample.

### 3.2 The adsorptive property of mercury

#### 3.2.1 Untreated MCM-41 and the modified MCM-41

From the Fig. 8a, it is shown that the adsorption rate of modified MCM-41 is higher than that of MCM-41. In order to investigate the different adsorption of mercury between MCM-41 and modified MCM-41, the batch experiments were carried out in 50 mL borosil conical flasks by agitating 0.01 g of MCM-41 and modified MCM-41 respectively with 10 mL of the aqueous mercury solution for 5 h at 303 K on a air-bath-mechanical shaker. The adsorption studies of MCM-41 and modified MCM-41 were carried out with different initial concentrations of mercury(II) from 2 to 14 mg/L while maintaining the adsorbent dosage at 0.01 g. The residual concentration of mercury in the solution was determined by ICP-AES.

**Fig. 7:**
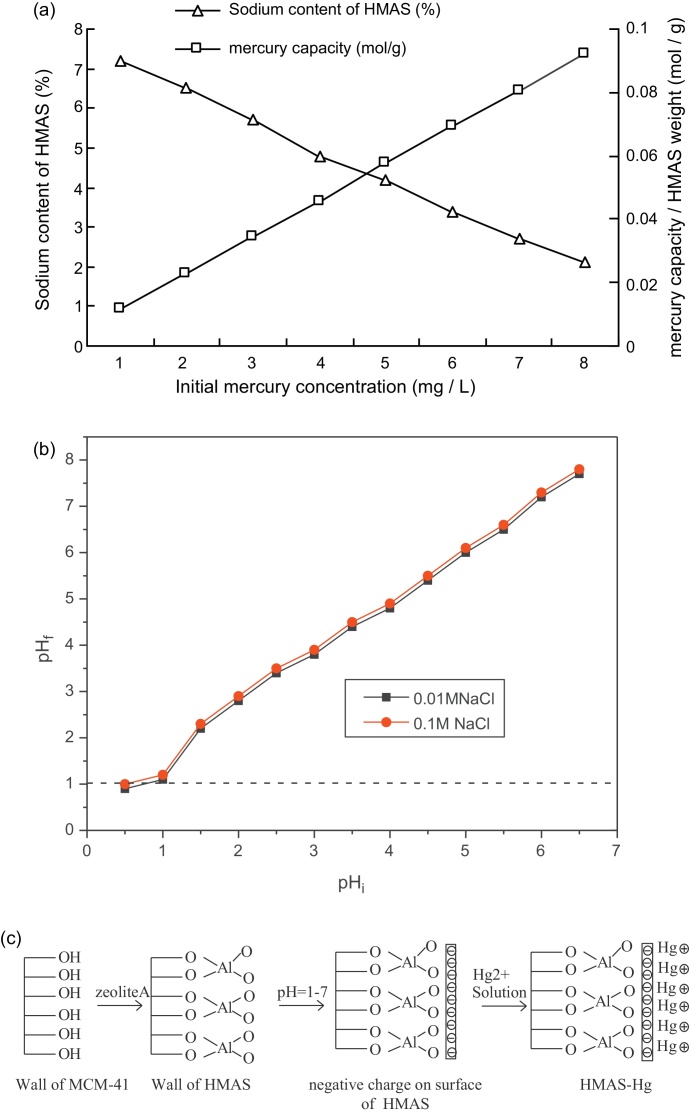
The sodium content of HMAS versus mercury capacity at different mercury concentration (a); pH_ZPC_ plots of adsorbents HMAS (b); proposed mechanism of zeolite A impregnated into MCM-41 and Hg(II) adsorption on HMAS (c).

The uptake of Hg(II) on HMAS was higher than that on MCM-41. The reason is analyzed from ^27^Al MAS-NMR and pH_ZPC_ of HMAS. As shown in Fig. 7a, the sodium content of HMAS decreased as mercury capacity increased. After zeolite A was impregnated into the porous walls of MCM-41, the unsaturated negative charge surface environment generated because some ions of Si^4+^ were replaced by Al^3+^ in the pore skeleton. According to the fundamental of ion exchange between solid and liquid phases, the ion exchange process between HMAS frame and aqueous mercury solution can be expressed by the following equation.

HMAS − 2Na^+^ + Hg^2+^ ↔ HMAS − Hg^2+^ + 2Na^+^

The untreated MCM-41 adsorbed mercury ion only through electrostatic interaction because of large surface area. The HMAS removed Hg^2+^ through both ion exchange and electrostatic interaction. Thus, the mercury removal rate of HMAS is much higher than that of original MCM-41.

Therefore, the following study was carried out with the modified MCM-41 as mercury adsorbent due to the high efficiency of mercury adsorption compared to the origin MCM-41.

#### 3.2.2 Effect of contact time

**Fig. 8:**
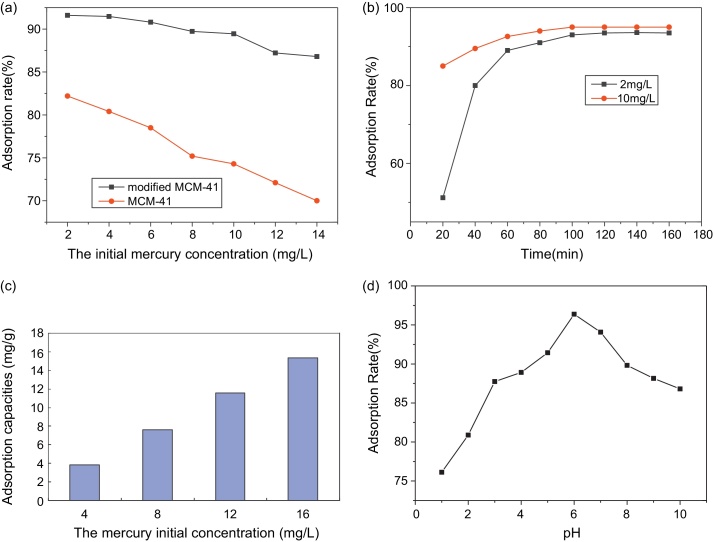
The adsorption rate of mercury at different initial mercury concentration using the MCM-41 and HMAS as adsorbent (a); the adsorption rate of HMAS at different adsorption time (*C*_0_ = 2 mg/L, *C*_1_ = 10 mg/L, pH 7, *m* = 1 mg/ml, temperature = 303 K) (b); Effect of initial mercury concentration on the adsorption of mercury (adsorbent mass *m* = 1 mg/ml, temperature = 303 K, the contact time: 5 h). (c); the effect of pH on adsorption of mercury (*C*_0_ = 10 mg/L, *T* = 5 h, *m* = 1 mg/ml, temperature = 303 K, the contact time: 5 h) (d).

The contact time affecting adsorption capacity of molecular sieve was studied at different initial mercury concentration. From the Fig. 8b, the optimum adsorption time is about 100 min. The removal of mercury was considerable after 100 min in the shaker and the equilibrium was also attained. Thus, the optimum contact time of 100 min could be considered for optimum adsorption of mercury on mesoporous molecular sieve. In addition, all following studies were carried out for 5 h duration due to obtain the optimum equilibrium results.

#### 3.2.3 The effect of initial mercury concentration

In order to study the certain amount of molecular sieve for the adsorption of mercury at different initial mercury concentration, the adsorption experiments were carried out by adding 10 mg molecular sieve to series flasks containing 10 mL of 2–16 mg/L of the initial mercury concentration. The measured results are shown in Fig. 8c. It showed that when the amount of molecular sieve was 10 mg, the adsorption percentage was kept above 90% at different mercury concentration. Besides it, the adsorption rate did not decrease with the mercury concentration increasing and the residual mercury concentration was below 0.6 mg/L. Thus, it also verified that this adsorbent is very effective for mercury adsorption. The maximum adsorption of mercury is 20 mg mercury/g adsorbent.

#### 3.2.4 Effect of pH

The pH of a solution is an important parameter affecting adsorption of metal ions on adsorbents. It is because it not only affects metal species in solution, but also influences the surface properties of adsorbents in terms of dissociation of functional groups and surface charge.

Solutions were prepared at different pH values ranging from 1.0 to 12.0 in order to determine the effect of pH on adsorption capacity of molecular sieve. The dependence of pH on the adsorption of mercury at an initial concentration of 10 mg/L onto molecular sieve is illustrated in Fig. 8d. It is evident that the adsorption capacities of mercury are affected by the pH ranging from 1.0 to 10.0. As shown in Fig. 8d, the solution pH had a significant effect on adsorption, and adsorption capacities were seen to increase with increased solution pH. The optimum pH value appeared to be about 6.0 for mercury removal. At lower pH (<6.0), Hg(II) was in the free ionic form of Hg^2+^, and the positively charged hydrogen ions may have competed with the Hg^2+^ for binding sites on the surface of the adsorbent [28]. Once the surface of adsorbent was protonated, the electrostatic interaction decreased. It was not beneficial for Hg^2+^ reaction with the surface of the adsorbent, resulting in lower adsorption capacities at lower pH. Besides it, the typical siliceous hexagonal structure of MCM-41 is destroyed above pH 8, which reduces the amount of mercury adsorption [30].

For adsorption onto the solid surface, six adsorption mechanisms might be supposed to exist (i.e., electrostatic interaction, ion exchange, ion–dipole interactions, coordination by surface metal cations, hydrogen bonding, and hydrophobic interaction [31]. The electrostatic interaction and ion-exchange mechanisms are responsible for the mercury adsorption because mercury is a metal cation. The ion–dipole interactions between the charged surface and the divalent cation are negligible in this experiment. Thus, the adsorption mechanisms could be summarized to the electrostatic interaction and ion-exchange mechanisms. The explanation for the fluctuation of mercury adsorption with the change of solution pH could be that the mesoporous sieve is relatively stable with high acid assistance. However, the surface charges changed in acid solution and decrease the electrostatic interaction. Additionally, its structure is destroyed in basic solution and it decreases the ion-exchange sites and electrostatic interaction.

The other important factor affecting Hg(II) removal is point of zero charge (pH_ZPC_) of the HMAS. The pH_ZPC_ determines the electrophoretic mobility where the net total particle charge is zero [30]. The pH_ZPC_ of HMAS was 1.0 from Fig. 7b. This implies that in mercury solution at any pH value above 1.0 the MCM-41 surface will bear a progressively increasing negative charge which is counterbalanced by the mercury cations. The physical meaning of the low pH_ZPC_ value is that the material is a very promising adsorbent for mercury removal in a wide pH range as it bears negative charge at pH > 1.0.

### 3.3 Adsorption isotherms

The adsorption isotherms of Hg(II) onto mesoporous molecular sieve were illustrated according to two parameter models, Langmuir and Freundlich and Redlich–Peterson model. Freundlich and Langmuir models are the most common isotherms for determining adsorption phenomena. Equilibrium data for mercury adsorption on molecular sieve were applied to Freundlich and Langmuir equations.

Freundlich model supposes that uptake or adsorption of metal ions occurs on the heterogeneous surface by monolayer adsorption. The equation of this model is described following like this:

(3-1) $$q_{\text{e}}=k_{\text{f}}{\left(c_{\text{e}}\right)}^{1/n}$$

The Freundlich equation can be linearized by taking logarithms and constants can be determined. The above equation can be linearized as follows:

(3-2) $$\log(q_{\text{e}})=\log\;k_{\text{f}}+1/n\log(c_{\text{e}})$$

Where $k_{\text{f}}$ and 1/*n* are Freundlich constants related to adsorption capacity and adsorption intensity, respectively. The initial concentrations of mercury were varied and adsorbent dose was kept constant in order to determine the equilibrium isotherms.

The Langmuir model assumes that uptake of metal ions occurs on a homogeneous surface by monolayer adsorption without interaction between adsorbed ions. The model is described in the following equation form:

(3-3) $$q_{\text{e}}=q_{\max}\frac{K_{\text{L}}C_{\text{e}}}{1+K_{\text{L}}C_{\text{e}}}$$

The above equation can be also linearized by the following process:

(3-4) $$\frac{1}{q_{\text{e}}}=\frac{1}{q_{\max}K_{\text{L}}}\cdot \frac{1}{C_{\text{e}}}+\frac{1}{q_{\max}}$$

where $q_{\text{e}}$ denotes the amount adsorbed at equilibrium and $q_{\max}$ is the Langmuir constant, which is equal to the adsorption capacity. The parameter $K_{\text{L}}$ represents the Langmuir adsorption equilibrium constant and $C_{\text{e}}$ is the equilibrium concentration.

**Fig. 9:**
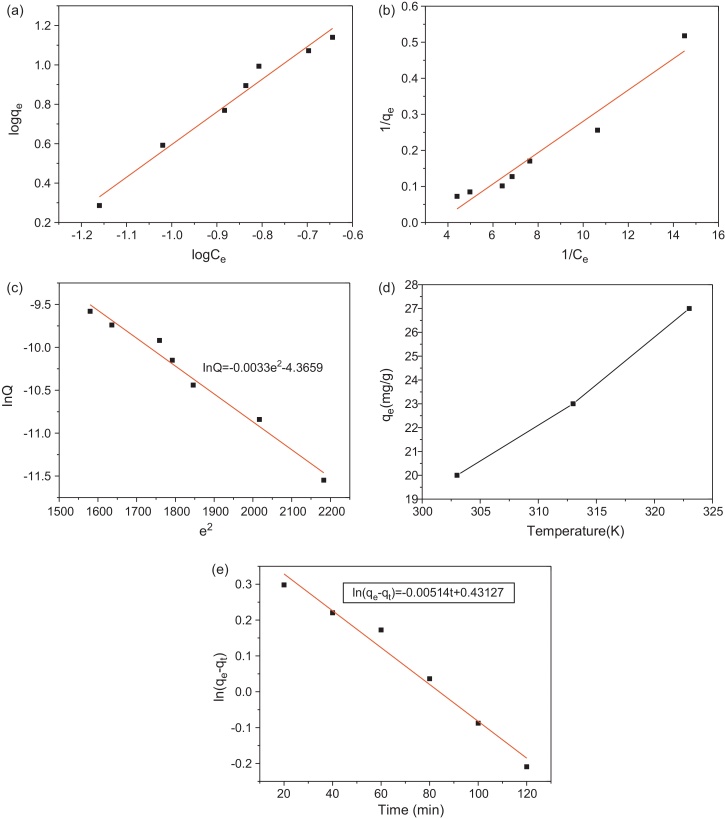
(a) The plot of Freundlich isotherm for adsorption of mercury on HMAS (adsorbent mass: 1 mg/ml, *T*: 303 K; initial concentration of mercury *C*_0_: 2–14 mg/L; adsorption time: 5 h; pH: 6.0). (b) The plot of Langmuir isotherm for adsorption of mercury(II) on HMAS (Initial concentration of mercury *C*_0_: 2–14 mg/L; adsorbent mass *m*: 1 mg/ml; adsorption time: 5 h; pH: 6). (c) The plot of Dubnin–Radushkevich isotherm for adsorption of mercury on HMAS (adsorbent mass: 1 mg/ml; temperature: 303 K; initial concentration of mercury: 2–14 mg/L; pH: 6.0). (d) The adsorption capacity of mercury at different temperature (*C*_0_ = 16 mg/L mercury, temperature = 303, 313, 323 K, pH: 6.0, adsorbent mass: 1 mg/ml). (e) The plot of first order pseudo equation for the adsorption of mercury on HMAS (*C*_0_ = 16 mg/L, adsorbent mass: 1 mg/ml, temperature: 303 K, pH: 6.0).

Plots of $\log\;C_{\text{e}}$ vs. log *q* and $1/C_{\text{e}}$ vs. $1/q_{\text{e}}$ evaluated the Freundlich and Langmuir isotherms of mesoporous sieve were shown in Fig. 9a and b, respectively.

The parameters for the Langmuir and Freundlich isotherms were evaluated based on the data from present experimental systems (Table 4), with the Freundlich fitting the data better than the Langmuir. This implies that mercury adsorption onto the HMAS is more like a monolayer adsorption process with heterogeneous distribution It seemed that the equilibrium adsorption capacity was distinctively improved by increasing temperature from 303 K to 323 K. It is because the effective adsorption sites on the adsorbent became more and mercury diffusion process may be promoted under higher temperature conditions in which mercury ion moves more quickly in solution. In addition, water viscosity decreases and adsorption sites on the adsorbent become more energetic.

Adsorption is considered to be satisfactory when the value of Freundlich constant *n* is the value between 1 and 10 [32]. Additionally, if the value of Freundlich isotherm constant *n* is between 0 and 2, it will suggest that the mesoporous sieve adsorbent has the uniform, monolayer and even distribution [33]. The data of *K*_f_ and *q*_max_ represent the adsorption capacity. The Freundlich and Langmuir isotherm model both provide well fitting to the equilibrium data.

Adsorption of mercury could also be explained by the Dublin–Radushkevich (D–R) isotherm. The adsorption mechanisms can be drawn from the following equation:

(3-5) $$InQ=InQ_{\text{m}}-ke^{2}$$

The Polanyi potential *e* is calculated from the equation $RTIn(1+1/C_{\text{e}})$. Where *R* is gas constant, 8.314 J mol^−1^ K^−1^; *T*: adsorption temperature, 303 K; and $C_{\text{e}}$: equilibrium concentration, mmol/L.

In the Eq. (3-5), *Q* denotes the amount adsorbed per unit mass of adsorbent (mol/g), *Q*_m_ is the adsorption capacity (mol/g), *k* is a constant related to the adsorption energy (mol^2^ kJ^−2^).

The D–R isotherm of *e*^2^ vs. *InQ* is shown as Fig. 9c. The adsorption energy that is denoted as *E* can be calculated using D–R equation and the equation is following like this:

(3-6) $$E={\left(-2\text{k}\right)}^{-0.5}$$

The value of *E* is 13.58 kJ/mol according to the Eq. (3-6). The adsorption process occurs by chemical ion exchange when *E* is between 8 and 16 kJ/mol. In addition, the adsorption is physical type if *E* is less than 8 kJ/mol [32]. Thus, the process of mercury adsorption involves the chemical ion-exchange mechanism.

### 3.4 Thermodynamic studies

The effects of temperature on adsorption rate of mercury on the adsorbent were studied at 303, 313, and 323 K. Fig. 9d shows the changeable trends of mercury adsorption with the increase in temperature. As shown in Fig. 9d, the mercury ions were favorably adsorbed from 20 mg/g to 27 mg/g with rise in temperature from 303 K to 323 K for the adsorbent at the mercury concentration of 16 mg/L and pH of 6.

The evolution of heat accompanied the mercury adsorption due to the mercury ions are stabilized on the adsorbent surface. The temperature dependency of Henry's constant ‘*k*’ obeys the van’t Hoff equation like this:

(3-7) log k=ΔS/2.303R−ΔH/2.303RT

If energy change is free, the following equation will be adopted,

(3-8) ΔG=−RTlnk

As shown in Table 5, the conclusion can be drawn that the adsorption process of mercury is spontaneous due to the negative values of ΔG. Besides it, the process is also the endothermic process because ΔH is the positive value. ΔS denotes that the feasibility and randomness at the adsorbent and solution interface during the adsorption process.

It seemed that the equilibrium adsorption capacity was obviously improved by increasing temperature from 303 K to 323 K. It is because the effective adsorption sites on the adsorbent became more and mercury diffusion process may be promoted under higher temperature conditions in which mercury ion moves more quickly in solution. In addition, water viscosity decreases and adsorption sites on the adsorbent become more energetic as temperature increasing.

### 3.5 Adsorption dynamics

The mercury uptake rate and the residence time of adsorbent are illustrated by different adsorption dynamic models. The adsorption dynamic models contain orders equations such as first-order pseudo, second-order pseudo and so on. The parameter *r* can present whether the data can be fitted into the adsorption model very well. If the *r* value is very high, it will show that the model successfully describes the kinetics of mercury adsorption.

The first-order pseudo equation

The adsorption dynamics can be determined using the following equation like this:

(3-9) $$dq/dt=K_{1}(q_{\text{e}}-q_{t})$$

where $q_{\text{e}}$ denotes adsorption capacity at equilibrium (mg/g); $q_{t}$ represents adsorption capacity at time *t* (mg/g) and *K*_1_ is first-order pseudo constant (min^−1^).

In order to calculate parameters of the Eq. (3-9), the rearrangement is obtained like this:

(3-10) $$\ln(q_{\text{e}}-q_{t})=\ln q_{\text{e}}-k_{1}t$$

The values of $\ln(q_{\text{e}}-q_{t})$ are linearly correlated with *t* according to the Eq. (3-10). The plot of $\ln(q_{\text{e}}-q_{t})$ versus time *t* is shown in Fig. 9e. The linear regression coefficient is about 0.98, which illustrates that the kinetics of mercury adsorption process fits well with first order kinetics of adsorption.

## 4 Conclusions

Adsorption of mercury on HMAS prepared with fly ash and modified by impregnation with zeolite A precursors was studied at different conditions. The HMAS showed very high efficiency for removing mercury(II) compared to the origin MCM-41. pH of solution, temperature and initial mercury concentration were studied for the efficiency of mercury adsorption. The highest adsorption capacity of HMAS happened at 323 K, and at pH 6. In addition, the equilibrium data fit well to Langmuir and Freundlich models. The R–D model also demonstrates that the process of mercury adsorption involved the chemical ion-exchange mechanism. The thermodynamic study illustrates that the adsorption process is endothermic with chemisorption mode. The kinetics of mercury adsorption also follows the first order pseudo kinetics. In conclusion, HMAS is a very effective adsorbent for mercury adsorption.

## Figures and Tables

**Table 1:** Fly ash mineralogical composition.

Fly ashes	Quartz (%)	Mullite (%)	Amorphous SiO_2_ (%)	Amorphous Al_2_O_3_ (%)
ZF-1	3.88	30.69	36.23	17.19
ZF-2	11.58	3.78	44.8	17.84

**Table 2:** Fly ash mineralogical composition after acid treatment.

Fly ashes	Quartz (%)	Mullite (%)	Amorphous SiO_2_ (%)	Amorphous Al_2_O_3_ (%)
ZF-1	3.88	30.69	51.23	2.19
ZF-2	11.58	3.78	60.14	2.5

**Table 3:** The concentration of silica and alumina in the alkali solutions after alkali treatment.

Fly ashes	SiO_2_ (mg/L)	Al_2_O_3_ (mg/L)	NaOH(mg/L)
ZF-1	25360	584.56	80
ZF-2	31520	23.1	80

**Table 4:** Freundlich and Langmuir constants and correlation coefficients for adsorption of mercury on HMAS.

Freundlich constants				Langmuir constants		
Sample	*K*_f_ (mg/g)	*n*	*R*^2^	*q*_max_ (mg/g)	*K*_L_ (L/mg × 10^3^)	R^2^
Mesoporous sieve	18.95	1.214	0.972	20.655	4.58	0.94

**Table 5:** The different values of parameters of the thermodynamic model.

Temperature (*K*)	ΔG (KJ/mol)	ΔH (J/K/mol)	ΔS (KJ/mol)
303	−10.65	25.8	150.13
313	−13.48		
323	−14.56		
